# Evolution and comparative ecology of parthenogenesis in haplodiploid arthropods

**DOI:** 10.1002/evl3.30

**Published:** 2017-11-09

**Authors:** Casper J. van der Kooi, Cyril Matthey‐Doret, Tanja Schwander

**Affiliations:** ^1^ Department of Ecology and Evolution University of Lausanne Lausanne Switzerland

**Keywords:** asexual reproduction, haplodiploidy, Hymenoptera, niche breadth, thelytoky, Thysanoptera, arrhenotoky, polyploidy

## Abstract

Changes from sexual reproduction to female‐producing parthenogenesis (thelytoky) have great evolutionary and ecological consequences, but how many times parthenogenesis evolved in different animal taxa is unknown. We present the first exhaustive database covering 765 cases of parthenogenesis in haplodiploid (arrhenotokous) arthropods, and estimate frequencies of parthenogenesis in different taxonomic groups. We show that the frequency of parthenogenetic lineages extensively varies among groups (0–38% among genera), that many species have both sexual and parthenogenetic lineages and that polyploidy is very rare. Parthenogens are characterized by broad ecological niches: parasitoid and phytophagous parthenogenetic species consistently use more host species, and have larger, polewards extended geographic distributions than their sexual relatives. These differences did not solely evolve after the transition to parthenogenesis. Extant parthenogens often derive from sexual ancestors with relatively broad ecological niches and distributions. As these ecological attributes are associated with large population sizes, our results strongly suggests that transitions to parthenogenesis are more frequent in large sexual populations and/or that the risk of extinction of parthenogens with large population sizes is reduced. The species database presented here provides insights into the maintenance of sex and parthenogenesis in natural populations that are not taxon specific and opens perspectives for future comparative studies.

**Impact Summary**

The animal kingdom exhibits a great diversity in reproductive modes. In addition to the well‐known and widespread sexual reproduction, species can reproduce asexually, via parthenogenesis. Males are absent in parthenogenetic species or populations. How this diversity in reproductive systems can be maintained remains a major question in evolutionary biology.

We assembled a database of parthenogenetic arthropod species focusing on groups with haplodiploid sex determination. Haplodiploidy is the sex determination system where males develop from unfertilized eggs and females from fertilized eggs. We use our database to identify ecological traits that contribute to reproductive polymorphisms.

Parthenogenesis evolved many more times than previously thought. We found clear evidence for parthenogenesis in 765 species in many phylogenetically unrelated groups with vastly different ecologies. The frequency of parthenogenesis greatly varies among groups, and many species comprise both sexual and parthenogenetic populations. Overall, the frequency ranges from 0–1.5% between orders, but in species‐rich genera, parthenogenesis occurs in up to as much as 38% of the species. Polyploidy is very rare (at most 4%), and endosymbiont‐induced parthenogenesis is suggested to occur in approximately 40% of the species. Parthenogens are characterized by larger, polewards extended geographic ranges and utilize more host species than their sexual relatives. Moreover, ecological attributes (i.e. the number of host species and size of geographic distribution) in sexuals favor the transition to and/or the success of derived parthenogenetic lineages. This species database sets the basis for further analyses on sexuals and parthenogens that are not taxon specific.

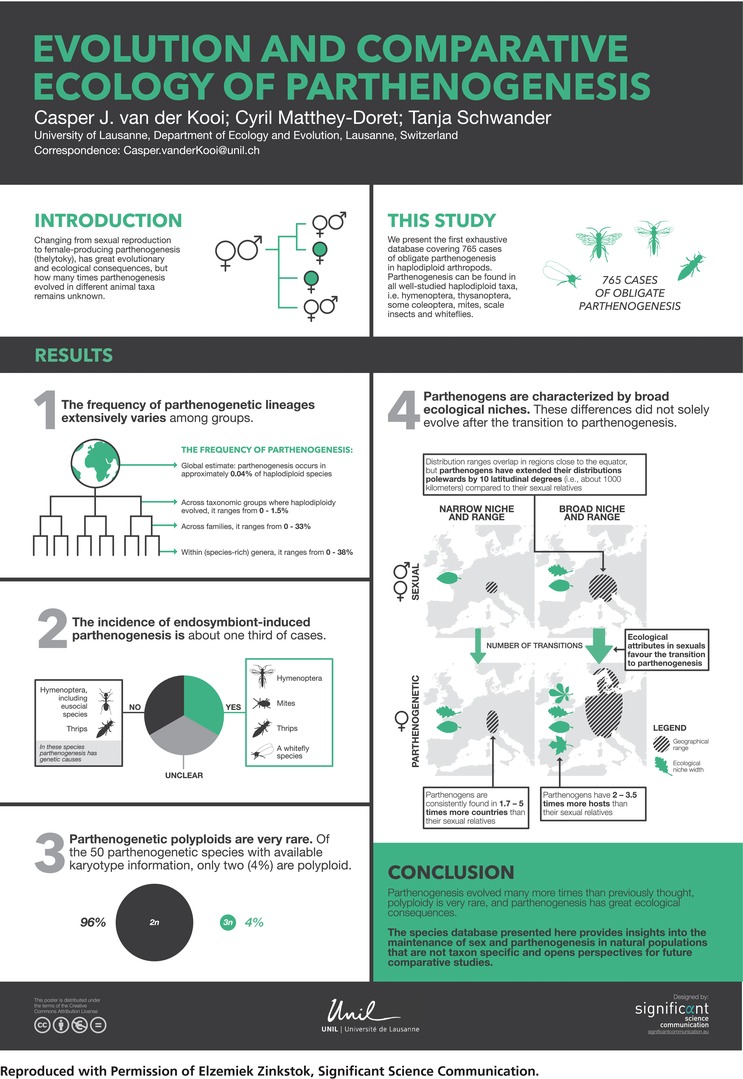


Changes in reproductive modes, especially from sexual reproduction to female‐producing parthenogenesis (also called thelytoky), have great evolutionary and ecological consequences (Bell 1982), but how many times parthenogenesis evolved in different animal taxa is unknown. Whereas parthenogenesis is rare amongst vertebrates and absent in natural bird and mammal populations, it occurs frequently in many species‐rich invertebrate groups (Bell 1982; Suomalainen et al. 1987; Normark 2003). Using information mostly from vertebrates, White (1977) estimated that approximately 0.1% of the described animal species reproduce by means of parthenogenesis, a frequency estimate that subsequently has been widely perpetuated (Bell 1982; Schön et al. 2009). This estimate was, however, not based on a species list, and vertebrates represent only a minute and nonrepresentative fraction (at most 1%; (May 2010)) of total animal diversity. More importantly, parthenogenesis in vertebrates is often due to tychoparthenogenesis (i.e., rare, spontaneous hatching of unfertilized eggs in sexual species), rather than facultative or obligate parthenogenesis. Given the inefficiency of tychoparthenogenesis (very low hatching success and low viability of offspring; reviewed by van der Kooi and Schwander 2015), it generally plays no role in the population dynamics of a species.

In contrast to vertebrates, the frequency of obligate parthenogenesis in some species‐rich invertebrate groups appears to be much higher than 0.1%. Focusing on hexapods, Normark (2014) recently pointed out that in some insect groups the overall frequency can be orders of magnitudes higher. Indeed, studies that focused on specific invertebrate groups found high frequencies of parthenogenesis, for example it was found in 15% of *Megastigmus* (Boivin et al. 2014) and 30% of *Aphytis* wasp species (DeBach 1969; Rosen and DeBach 1979). Why different taxa vary by several orders of magnitude in the frequency of parthenogenesis remains unknown.

One hypothesis that could explain the extensive variation in parthenogenesis frequency among taxa is that developmental and genetic constraints reduce the transition from sexual reproduction to parthenogenesis in some cases (reviewed by Engelstaedter 2008). For example, the necessity of sperm to initiate embryo development could explain the extreme rareness of parthenogenesis in vertebrates. In many invertebrates such developmental requirements are absent. As an example, in species with haplodiploid sex determination–where males develop from unfertilized eggs and females from fertilized eggs (arrhenotoky)—egg activation and centrosome formation is induced immediately after oviposition, independent of fertilization. However, within taxonomic groups with identical sex determination systems the incidence of parthenogenesis also varies (Normark 2003). This suggests that alternative factors, possibly linked to species ecologies rather than to genetic or developmental factors, also influence the frequency of parthenogenesis. The lack of quantitative frequency estimates of parthenogenetic species however prohibits testing how genetic and ecological effects influence the transition rate to parthenogenesis and the persistence of parthenogenetic lineages across broad taxonomic groups.

Here, we present the first comprehensive survey of female‐producing parthenogenesis in haplodiploid arthropods. Haplodiploid sex determination has evolved at least 17 times independently, of which 15 times in arthropods (Otto and Jarne 2001). This allows for comparative analyses across different taxonomic groups but within a single sex‐determination system. Furthermore, approximately 12% of all animal species are haplodiploid (Bachtrog et al. 2014), and the ecologies of several haplodiploid taxonomic groups (notably insects) are well studied. In arthropods, haplodiploidy is well known from the insect orders Hymenoptera (ants, bees, sawflies, and wasps) and Thysanoptera (thrips) where all species are haplodiploid, but it also occurs in Hemiptera (whiteflies), a few beetle species and several groups of mites. We focus on obligate female‐producing parthenogenesis, thus excluding rare cases of facultative and cyclical parthenogenesis, as these reproductive systems are functionally very similar to sexual reproduction (reviewed by Neiman et al. 2014). We do not include species characterized by paternal genome elimination–where males develop from fertilized eggs, but subsequently eliminate their paternal chromosomes, which has recently been reviewed elsewhere (e.g., Ross et al. 2010). Whenever possible, our database includes information on the causes of parthenogenesis in a lineage, especially whether parthenogenesis is caused by infection with maternally inherited endosymbionts (e.g., *Wolbachia*). Endosymbiont infection is a common cause of parthenogenesis in various haplodiploid groups (Stouthamer et al. 1990; Zchori‐Fein et al. 2001). For each taxonomic group, we calculate frequency estimates of parthenogenesis, endosymbiont‐induced parthenogenesis, and polyploidy, and we perform comparative tests on ecological characteristics of sexuals and parthenogens.

## Materials and Methods

### DATA COLLECTION

The species list was compiled via a thorough search through Google Scholar (publications until August 2016) and using previously published reviews on different topics (Table 1). We started with the list provided by Normark (2003) that contained 163 cases of parthenogenesis in haplodiploid taxa, which is about 20% of our database. Other (often overlapping) studies were used to extend our database (Table 1), and various overviews provided a starting point for searches in specific taxa (e.g., Lewis 1973; Cook and Butcher 1999; Wenseleers and Billen 2000; Huigens and Stouthamer 2003; Koivisto and Braig 2003). The recently established Tree of Sex database (Bachtrog et al. 2014) also included parthenogenetic species in various taxa, but did not list any parthenogenetic Hymenoptera or Thysanoptera species. All cases that were obtained from reviews were re‐examined.

**Table 1:** Important previous overview studies with parthenogenetic haplodiploids

Taxa studied	Species	Reference
Hemiptera, Hymenoptera, and Thysanoptera	163	(Normark 2003)
Hymenoptera	20	(Flanders 1945; Slobodchikoff and Daly 1971; Gokhman 2009)
Hymenoptera	100	(Stouthamer 2003)
Hymenoptera: Cynipoidea	50	(Askew et al. 2013)
Hymenoptera: *Aphytis*	30	(Rosen and DeBach 1979)
Mites	38	(Norton et al. 1993)
Thysanoptera	46	(Pomeyrol 1929)
*Haplodiploid arthropods*	*765*	*This study*

Note that different species lists often overlap.

Reproductive modes in most studies are deduced based on breeding experiments or population sex ratios. Species for which reproductive modes were not assessed via breeding assays were only included if sex ratio estimates were based on large sample sizes, preferably from different locations; hence, we are fairly certain that the species in our list are parthenogenetic. Species not present in the list were assumed to be sexual. However, for the vast majority of these, the reproductive mode has not been studied, and therefore our frequency estimates are underestimates. When available, information on ploidy levels, reproductive polymorphisms, life‐history traits as well as the origin and cytological basis of parthenogenesis was included in the database.

Frequencies of parthenogenesis are compared for different taxonomic levels (genera, families, superfamilies, and orders). Total species numbers for each taxonomic level were taken from large‐scale overview studies on Hymenoptera in general (Aguiar et al. 2013), Symphyta (Taeger et al. 2010; Taeger and Blank 2011), Chalcidoidea (Noyes 2016), and Thysanoptera (Mound 2013; ThripsWiki 2016).

### COMPARATIVE ANALYSES

For our comparative analyses of sexuals and parthenogens, we focused on the mega‐diverse Hymenoptera superfamily Chalcidoidea, taking advantage of the many transitions to parthenogenesis within this group (233 parthenogenetic species) as well as the availability of detailed ecological and taxonomic data in the Universal Chalcidoidea Database (Noyes 2016). Species with reproductive polymorphisms were excluded from these analyses, as separate information for sexual and parthenogenetic lineages within such species was generally not available.

We compared body size, number of host species [a proxy of a species’ niche breadth (Jaenike 1990)] and geographic distribution. Whenever possible given the available data, we compared sexuals and parthenogens via two ways: first, parthenogens were compared with their sexual sister‐species as deduced from recently published phylogenies (Table 2). In this comparative approach we included 44 parthenogens and 74 sexuals, belonging to eight genera in the families Aphelinidae, Torymidae, and Trichogrammatidae. These species were repartitioned into 32 sexual‐parthenogenetic pairs; for clades with multiple species, the mean value was used in the analyses. Second, information on number of host species and geographic distribution was automatically extracted from the Universal Chalcidoidea Database (Noyes 2016) using a custom Python script using BeautifulSoup4 (https://www.crummy.com/software/BeautifulSoup/). This approach allowed us to compare data from sexual and related parthenogens within 52 genera (comprising 134 parthenogens and 8194 sexual species. For the comparisons with species pairs, body size was obtained from taxonomic keys and scientific books or articles as sources. The size was measured in millimeters, as a single value or as a range, and excludes ovipositor length (see Supplementary Material for further details). Information on the number of host species used was mostly obtained from Noyes (2016), except for 13 species for which more recent data was available.

**Table 2:** Comparative analyses on morphological and ecological traits

Approach	Families	Genera	Pairs	Sexual	Parthenogen	Variables
Species pairs	3	8	32	74	44	Body size, host species, geographic distribution
Per genus	11	52	52	8194	134	Host species, geographic distribution

For the per genus analyses, we compared the number of host species and geographic distribution, which were obtained from Noyes (2016). To obtain accurate geographic distribution data in this approach, we replaced redundant location names by currently used names (e.g., Russia instead of USSR) and the large countries Australia, Brazil, Canada, China, India, Russia, and the USA were divided into states or regions. To transform country names to geographical information, we took the geographical center for a country (using OpenStreetMap Contributors, https://www.openstreetmap.org), using the Python package Geocoder (https://github.com/DenisCarriere/geocoder). The extreme latitude values were extracted using a custom python script (available at: https://github.com/cmdoret/chalcid_comparative_analysis).

## Frequency of Parthenogenesis

### PARTHENOGENESIS EVOLVED IN MANY DIFFERENT TAXONOMIC GROUPS

Cases of parthenogenesis can be found in all well‐studied haplodiploid taxa (Table 3). In total, we found evidence for obligate parthenogenesis in 765 species across nine orders, 33 superfamilies, 58 families, and 316 genera, which is about five times the number of species previously reported by Normark (2003) and many times the number of species in other reviews (Table 1). Although information on the phylogenetic relationships among different parthenogens is not available for most taxa, the 765 parthenogenetic species most likely correspond to at least as many independent transitions from sexual reproduction to parthenogenesis. Speciation after the transition to parthenogenesis is considered to be very rare (Bell 1982), and so are reversals to sexuality (van der Kooi and Schwander 2014b). Moreover, what is considered a single parthenogenetic species often corresponds to a pool of parthenogens that derive from multiple, independent transitions to parthenogenesis (e.g., Janko et al. 2008; van der Kooi and Schwander 2014a).

**Table 3:** Frequency of parthenogenesis in haplodiploid taxa

Orders	Common name	Parthenogens	Total species	Proportion	Species total reference
Astigmata	Mites	3	5000	0.001	(Norton et al. 1993)
Coleoptera: Micromalthidae	Telephone‐pole beetle*	1	1	1	(Normark 2003)
Coleoptera: Scolytinae	Bark beetles	1	4500	0.000	(Farrell et al. 2001)
Hemiptera: Aleyrodoidae	Whiteflies	4	1556	0.003	(Martin and Mound 2007)
Hemiptera: Margarodidae	Scale insects	3	428	0.007	(García Morales et al. 2016)
Hemiptera: Diaspididae	Scale insects	1	2378	0.000	(García Morales et al. 2016)
Hymenoptera	Ants, bees, sawflies and wasps	586	150,000	0.004	(Mayhew 2007)
Mesostigmata	Predatory mites	43	5000	0.009	(Norton et al. 1993)
Prostigmata	Mites	6	14,000	0.000	(Norton et al. 1993)
Thysanoptera	Thrips	91	5938	0.015	(Mayhew 2007)
Trombidiformes	Mites	26	25,821	0.001	(Zhang et al. 2011)

In mites and Scolytinae, the exact origin(s) of haplodiploidy are not known, so the higher taxonomic level was chosen. ^*^The telephone‐pole beetle, *Micromalthus debilis*, is the only extant species in this monotypic family, which is considered to have a haplodiploid origin, see Normark (2003).

Only taxa with at least one case of parthenogenesis described are shown.

**Figure 1:**
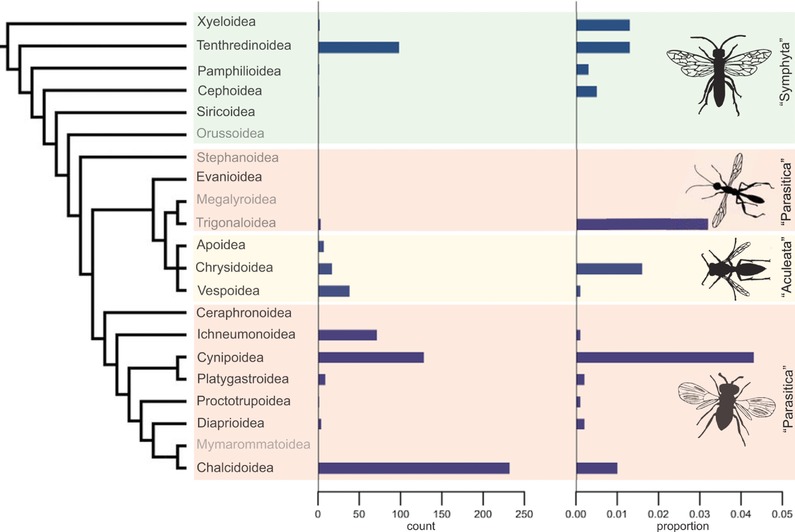
Frequency of parthenogenesis in Hymenoptera superfamilies. The phylogeny is from Klopfstein et al. (2013); taxa with fewer than 100 species described have gray label names. Except for the Ceraphronoidea, Evanioidea, and Siricoidea, all species‐rich taxa have parthenogenetic species.

The analysis of parthenogenesis frequencies at different taxonomic levels in haplodiploids indicates that different sex determination systems are not required to generate variation in parthenogenesis frequency among taxa. Indeed, there is extensive variation in parthenogenesis frequency among haplodiploid taxa and no phylogenetic clustering above the genus level. Overall, when excluding the exceptional case of the parthenogenetic *Micromalthus debilis* (the sole extant species in an ancient family), the frequency of parthenogenesis ranges from 0 to 1.5% across taxonomic groups where haplodiploidy evolved (Table 3). The global estimate of parthenogenesis across these groups is approximately 0.04%. Within the two largest insect clades (Hymenoptera: about 140.000 described species, Thysanoptera: 5000 described species; (Mayhew 2007)) the frequency of parthenogenesis ranges from 0 to 33%, with a mean of 0.9% among families in Hymenoptera and across Thysanoptera families from 0 to 3.7%, with a mean of 0.8%. Parthenogenesis is scattered across the whole phylogeny in hymenopterans and thrips (Figs. 1, S1–S3).

Some groups comprise particularly many parthenogenetic species. For example, Cynipoidea (the superfamily of gall wasps) stand out because they feature a very high frequency of parthenogenetic species. Approximately 4% of species (*n* = 122) are obligately parthenogenetic (Fig. 1). The high frequency of parthenogenesis is robust, because when the cases based on circumstantial evidence for parthenogenesis are excluded, the average frequency of parthenogenesis in gall wasps is still 1.3% (*n* = 38 species), which is markedly higher than in other superfamilies. Sexual gall wasps have a cyclically parthenogenetic life cycle, where a parthenogenetic cycle is generally followed by a sexual cycle (reviewed by Stone et al. 2002). The high number of obligate parthenogens in this system may be explained by simple loss‐of‐function mutations that suppress the sexual cycle (Neiman et al. 2014). As Cynipoidea is the only known haplodiploid arthropod group with cyclical parthenogenesis, formal tests to infer whether cyclical parthenogenesis favors the transition to obligate parthenogenesis are impossible.

Other groups stand out because of their high frequency of parthenogenesis. When genera with very few species are excluded, the Chalcidoidea genera *Aphytis* and *Trichogramma* feature a high frequency of parthenogenesis. In line with earlier estimates (DeBach 1969; Rosen and DeBach 1979), the highest proportion of parthenogens was found in the parasitoid wasp genus *Aphytis* s.l. where parthenogenesis occurs in 42 out of 110 (38%) species. In *Trichogramma*, 27 out of 239 species (11%) have parthenogenetic lineages. The reason for the high incidence of parthenogenesis in *Aphytis* is unknown, but for *Trichogramma* it is presumably due to ascertainment bias; in this genus the first cases of endosymbiont‐induced parthenogenesis were described (Stouthamer et al. 1990). This may have stimulated researchers to study reproductive systems in this genus. Similar frequencies were found among thrips genera (Supporting file 5). In summary, parthenogenesis is found in all major haplodiploid groups and the frequency greatly varies between taxonomic groups (Table 3; Figs. 1, S1–S3).

### REPRODUCTIVE POLYMORPHISMS ARE WIDESPREAD

In many plant and animal species both sexual and parthenogenetic lineages can be found, either sympatrically or in different geographic areas (Bell 1982; Lynch 1984). Such reproductive polymorphisms are interesting, as they can be used to study possible costs and benefits of sex under natural conditions. In our survey, we found that for 143 parthenogenetic haplodiploid species (19% of the parthenogenetic species in our study) there is clear evidence for existence of sexual lineages as well. Reproductive polymorphisms occur frequently across many taxonomic levels and at comparable frequencies in species with parthenogenesis caused by genetic factors or endosymbiont infection (respectively 10/27 species 37%, and 26/58 species 45%; **χ** ^2^ = 0.21, *df =* 1, *P* = 0.65). This suggests that the ecological and/or evolutionary factors that maintain reproductive polymorphisms seem similar for species with different causes of parthenogenesis. How frequently reproductive polymorphisms occur in other (nonhaplodiploid) taxonomic groups remains unknown, as there currently are no estimates for other taxa.

The frequency of reproductive polymorphisms is most likely underestimated. Species that do not occur in our database are assumed to be sexual, and parthenogenetic species for which no sexual lineages are known are considered to be parthenogenetic. Considerable research effort (sampling of populations across the species distribution range and breeding experiments) is required in order to detect reproductive polymorphisms. That increasing research effort may increase the chance of detecting reproductive polymorphism becomes clear when the number of citations for species with reproductive polymorphisms is compared with that for obligate parthenogens. Species with reproductive polymorphisms have more than twice as many citations as obligate parthenogens (Supplementary Material). An alternative, nonmutually exclusive explanation is that reproductive polymorphic species are more studied because of their polymorphism. We currently cannot formally test the two hypotheses; it nonetheless is likely that many sexual lineages in putatively parthenogenetic species as well as parthenogenetic lineages in putatively sexual species remain undetected.

## Origins of Parthenogenesis

### ENDOSYMBIONT‐INDUCED PARTHENOGENESIS IS PROVEN TO OCCUR IN 42% OF SPECIES

Transitions from sex to parthenogenesis can have different causes. Parthenogenesis can have a genetic basis, such as a hybridization event between related species or a mutation in sex‐specific genes, which may result in the origin of a parthenogenetic lineage (Normark 2003). Transitions to parthenogenesis can also be caused by infection with maternally inherited endosymbionts (Stouthamer et al. 1990). In haplodiploids, at least three taxa include parthenogenesis‐inducing endosymbionts (PI‐endosymbionts): *Wolbachia, Cardinium*, and *Rickettsia* (e.g., Stouthamer et al. 1993; Zchori‐Fein et al. 2001; Hagimori et al. 2006). Presence of PI‐endosymbionts can be tested by removing endosymbionts from parthenogenetic females, either by exposing them to high temperatures during early development (Flanders 1945) or by treating them with antibiotics (Stouthamer et al. 1990). This “cures” parthenogenetic females from their endosymbionts, and causes them to produce sons.

**Figure 2:**
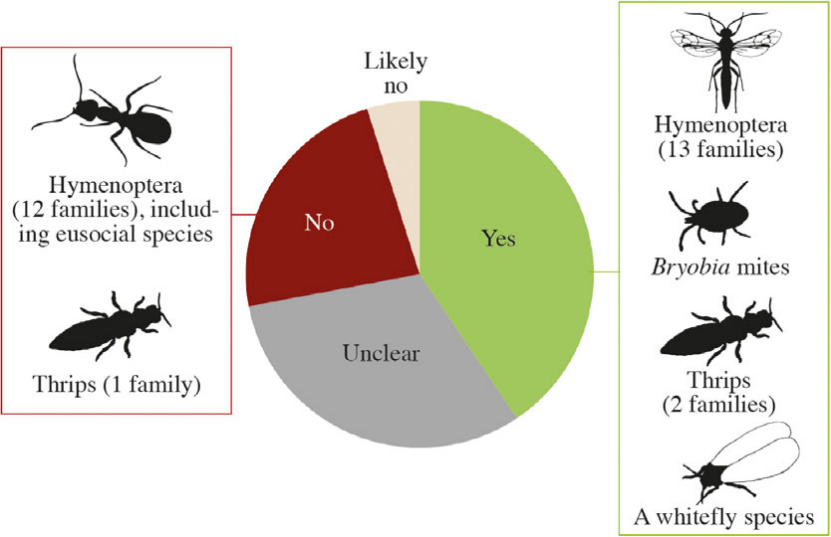
Frequency of endosymbiont‐induced parthenogenesis. Yes: sexual reproduction can be restored via antibiotic treatment and/or after exposure to heat. No: sexual reproduction cannot be restored and is not caused by endosymbionts. Unclear: only circumstantial evidence points to endosymbionts (e.g. only PCR screening and no antibiotic treatment; see main text). Likely no: no indication of parthenogenesis‐inducing endosymbionts, but reversibility to sexuality has not been tested. Based on 143 cases.

The frequency of endosymbiont‐induced parthenogenesis in haplodiploids is surprisingly high. Out of 139 species for which we obtained information on the causes of the transition to parthenogenesis, for 105 species it was suggested that parthenogenesis was caused by endosymbionts. However, in only 58 cases (42% of the investigated species) this was convincingly shown (Fig. 2). Clear cases of endosymbiont‐induced parthenogenesis are currently known in three insect orders (Hemiptera, Hymenoptera, and Thysanoptera) and in *Bryobia* mites (Table S3) (Weeks and Breeuwer 2001).

Three factors may generate an overestimate of the frequency of endosymbiont‐induced parthenogenesis. First, publication bias will skew the frequency of endosymbiont‐induced parthenogenesis toward positive results, as studies showing PI‐endosymbionts to be present are much more likely to be published than studies that show that PI‐endosymbionts are absent in a species [“negative results”; see (Monti et al. 2016) for a notable exception]. Second, infection with *Wolbachia* is often interpreted as evidence for an endosymbiont inducing parthenogenesis in a lineage (e.g., Weeks and Breeuwer 2001; Huigens and Stouthamer 2003; Boivin et al. 2014). These interpretations should be considered with caution, however, because *Wolbachia*‐infection is very widespread, including in many sexual species (Zug and Hammerstein 2012). Third, when parthenogenesis‐inducing endosymbionts have been found in a species, they are often assumed to occur in related parthenogenetic species, which is not necessarily true. Mechanisms underlying parthenogenesis can greatly differ between highly related species, as shown in *Trichogramma* (Vavre et al. 2004) and *Encarsia* parasitoids wasps (Gokhman 2009) as well as *Aptinothrips* grass thrips (van der Kooi and Schwander 2014a), where parthenogenesis is caused by endosymbionts in some species, but via another mechanism in others. In conclusion, in 42% of the investigated haplodiploid species parthenogenesis is due to endosymbionts, but the actual frequency presumably is much lower.

In many cases the endosymbiont species causing parthenogenesis remains unknown. *Cardinium* and *Rickettsia* have been shown to cause parthenogenesis in Chalcidoidea (Hymenoptera) only. Strong correlations with *Rickettsia* and parthenogenesis are found in three Eulophidae species, and *Cardinium* is suggested to cause parthenogenesis in several species of Aphelinidae and Encyrtidae. In the majority of systems *Wolbachia* is considered to be the causal endosymbiont, but this view is most likely biased because the other endosymbiont species were described more than a decade later. Consequently, numerous studies screened parthenogens for presence of *Wolbachia*, but did not screen for the other PI‐endosymbionts *Cardinium* and *Ricketsia*. This lack of complete screens, together with the fact that some parthenogenetic lineages are known to harbor more than one endosymbiont species (e.g., in *Aphytis* parasitoid wasps with PI‐endosymbionts, both *Cardinium* and *Wolbachia* are found (Zchori‐Fein and Perlman 2004)), highlight that we should be cautious in ascribing parthenogenesis to a specific endosymbiont species.

### POLYPLOIDY IS EXCEEDINGLY RARE

In vertebrates and plants, polyploidy occurs more frequently in parthenogens than sexuals (Suomalainen et al. 1987; Otto and Whitton 2000); as a consequence, polyploidy is sometimes considered the norm in parthenogens (e.g., Kearney et al. 2009). At least in haplodiploids this is not the case. Of the 50 parthenogenetic species with available karyotype information, only two (4%) are polyploid (Fig. S4), namely the gall wasp *Diplolepis eglanteriae* and the sawfly *Pachyprotasis youngiae* (both triploids) (Sanderson 1988; Naito and Inomata 2006). The frequency of polyploids drops even further when species with parthenogenesis‐inducing endosymbionts are included. Parthenogenesis‐inducing endosymbionts are unlikely to occur in polyploids, because endosymbiont‐induced parthenogenesis generally involves meiotic parthenogenesis with secondary restoration of diploidy. Including the 48 species with parthenogenesis‐inducing endosymbionts (but for which no karyotypes are known), the frequency of polyploid parthenogens drops to 2%. Objective frequency estimates of polyploidy in sexuals are lacking for any haplodiploid group, though it is thought to be rare in hymenopterans (Gokhman 2009). Nonetheless, given there are many parthenogens in haplodiploid groups, and very few of these are polyploid, polyploidy is certainly more rare among parthenogens than commonly thought.

## Ecological Differences between Sexuals and Parthenogens

Several hypotheses predict that sexual and parthenogenetic species differ in ecological generalism and the size of distribution ranges, with opposite predictions depending on the hypothesis (Vrijenhoek 1979; Bell 1982; Lynch 1984). For example, parthenogens might have broader ecological niches, because there may be lineage‐level selection for general‐purpose genotypes in parthenogens (Lynch 1984). Alternatively, parthenogens might have narrower niches, because when a parthenogen derives from a sexual ancestor it inherits a single genotype from a genetically heterogeneous sexual group (the “frozen niche variation hypothesis,” sensu Vrijenhoek 1979; see also Bell 1982).

We compared the number of host species and distribution ranges for obligate sexual and obligate parthenogenetic wasps in parasitoid and phytophagous wasps in the mega‐diverse Hymenoptera superfamily Chalcidoidea. Chalcidoidea was chosen because of the many independent transitions to parthenogenesis in this group (*n* = 233; Fig. 1) and because their ecology and geographic distribution are well‐documented (see Noyes 2016). We consider the number of host species as a proxy of a species’ niche width, that is, generalists will have more hosts than specialists (Jaenike 1990). Sexuals and parthenogens were compared in two different datasets. First using sexual‐parthenogenetic sister‐species pairs as inferred from phylogenetic trees (32 sexual‐parthenogen species pairs) and second via a coarser approach where data from all parthenogens and sexuals was compared per genus (8328 species in 52 genera). The data subsets used for comparisons of specific ecological traits depended on the availability of ecological and phylogenetic information (Materials and Methods, Supplementary Information).

**Figure 3:**
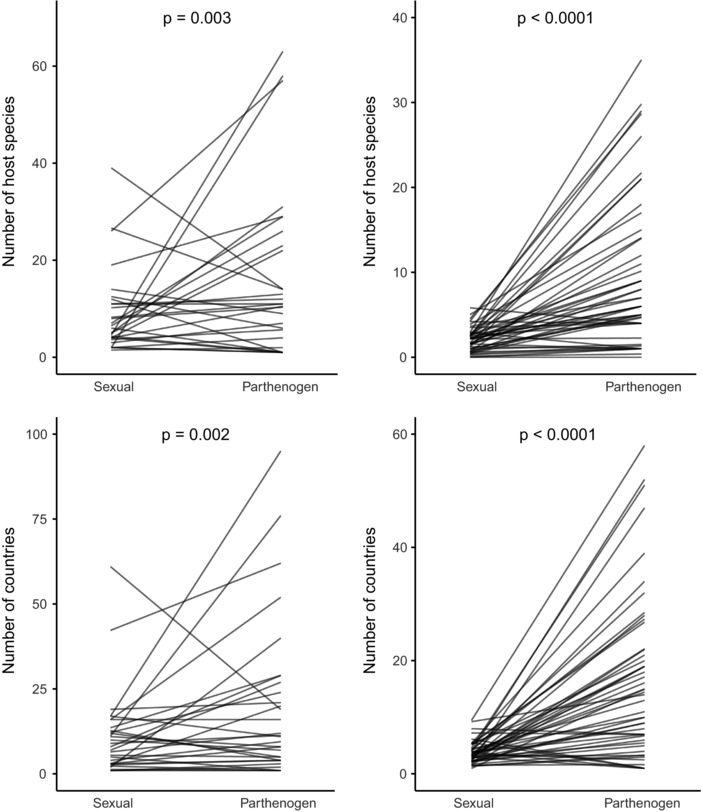
Number of host species for sexual and parthenogenetic Chalcidoidea parasitoid and phytophagous wasps. Left panels: pairwise analysis, right panels: analysis incorporating all data from the database; note the different y‐axis ranges. For visualization purposes, one pair with a parthenogen with extremely many host species is not shown in the upper panels.

Parthenogens consistently parasitize more host species, indicating they have wider ecological niches than sexuals. A significant 2–3.5‐fold increase in number of host species used was found via both the species‐pair and per genus approach (Fig. 3), and these results were robust with respect to publication bias (i.e., more hosts known for more intensively studies species; see Supplementary Information). Broad niches in parthenogens can stem from two mechanisms: few successful genotypes that have a broad ecological niche (i.e., “general purpose genotypes”) or a large mixture of genetically different clones each with a distinct and narrow ecological niche (Bell 1982; Lynch 1984). To disentangle these hypotheses, detailed studies on the genetic vs. niche diversity of parthenogenetic lineages are required.

Animals with a wider ecological niche are more likely to expand their geographic range (Normark and Johnson 2011). In line with this theory, we found that parthenogens occur in larger geographic regions than their related sexual species. Parthenogens are consistently found in 1.7–5 times more countries than their sexual relatives (Fig. 3). Distribution ranges of sexuals and parthenogens largely overlap in regions close to the equator, but parthenogens have extended their distributions polewards by 10 latitudinal degrees (i.e., about 1000 kilometers) compared to their sexual relatives (Fig. S5). These effects are robust with respect to publication bias (more occurrences known for more intensively studied species; see Supplementary Material).

**Figure 4:**
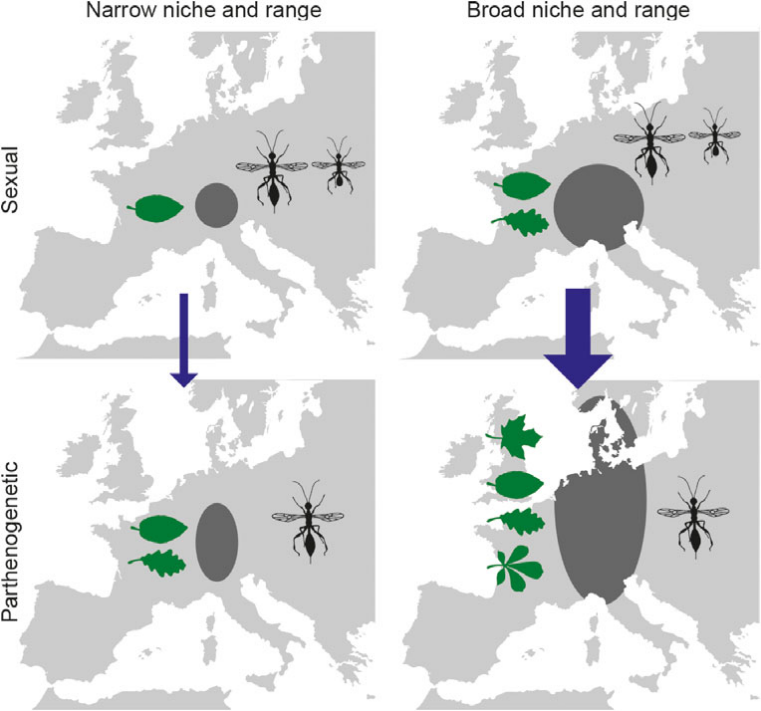
Parthenogens have a wider niche and polewards extended distribution as compared to sexuals, and parthenogenesis is more likely to evolve in sexuals with relatively wide niches and distribution ranges. Width of the arrow indicates the number of transitions, leaves represent the ecological niche width and dark shading represents geographical range.

The observed ecological and geographical differences between sexuals and parthenogens can stem from two nonmutually exclusive mechanisms. First, selection for a broad ecological niche and large geographical range can follow after the transition to parthenogenesis. Second, it is possible that transitions to parthenogenesis are more likely to occur in sexual species with broad niches or geographic distributions than in sexuals with narrow niches and distributions. To distinguish between these two scenarios, we compared the ecology and distribution ranges for two groups of sexuals: sexuals that are the sister‐species for a parthenogenetic species or clade, that is, the sexuals that likely share a common ancestor with the parthenogen, were compared with sexuals for which no related parthenogenetic species is known (the outgroup). We found that sexual species that share an ancestor with parthenogenetic species have more host species and occur in larger geographic areas than sexuals from clades where no parthenogenetic species are known (Fig. S6). This means that the increased niche width and enlarged geographic distribution (partly) arose before the transition to parthenogenesis and that (successful) parthenogenetic species arise more frequently from sexuals with wide ecological niches and geographic distributions, than from sexuals with narrow ecologies and geographic distributions (Fig. 4).

## General Discussion

Many isolated studies examined the frequency, mechanism, ploidy, and/or ecology of parthenogenesis in one or a few species (e.g., DeBach 1969; Vrijenhoek et al. 1989; Stouthamer et al. 1990; Boivin et al. 2014; van der Kooi and Schwander 2014a; Monti et al. 2016), but large‐scale, quantitative studies that provide insights into general patterns of parthenogens are lacking. We here present the first broad comparison on the ecology and evolution of parthenogenesis that is based on a species database that is as exhaustive as possible given the available data. Focusing on haplodiploid arthropods, we found parthenogenesis in more than 750 species across all major haplodiploid groups (ranging from 0 to 1.5% between orders; Table 3). In many phylogenetically different groups parthenogenesis occurs much more frequently than previously thought; as an example, in species‐rich Hymenoptera and Thysanoptera genera the frequency of parthenogenesis ranges from 0 to 38%. The absence of phylogenetic clustering above the genus level as well as the observation that parthenogenetic species are found in 2–3% of the genera (Supplementary Information) are very similar to frequency estimates of (facultative) asexual seed production in plants, which was found to occur in about 2.2% of phylogenetically distinct plant genera (Hojsgaard et al. 2014). However, the relative frequency of facultative versus obligate parthenogenesis in plants remains unknown. Our results are of great importance to our overall understanding of reproductive system evolution, as haplodiploidy is the sex determination system of many animal taxa (Bachtrog et al. 2014), including Hymenoptera, which is one of the most species‐rich insect groups (Mayhew 2007).

The number of obligate parthenogens in our database and the frequency estimates calculated here are certainly underestimates, as many cases of parthenogenesis remain undetected. For instance, in poorly studied species, female‐biased sex ratios will often remain unnoticed. More importantly, we assumed that species that do not occur in our list are by default sexual, while the reproductive mode of these species has not been directly investigated. Many species contain both sexual and parthenogenetic lineages (see section on reproductive polymorphisms), which may easily co‐occur within populations. Unless studied in detail via breeding assays, such mixed populations will generally be considered sexual, because they comprise males.

It is unlikely that the parthenogenesis frequency we report here is specific for haplodiploids, although addressing this question requires development of detailed species lists for other groups. In organisms with other sex‐determination systems, developmental processes, such as egg activation and centrosome formation, are supposed to impose constraints on the transition to parthenogenesis (Engelstaedter 2008). In haplodiploids, where males always develop parthenogenetically, several of these developmental constraints are overcome. For example, in haplodiploids egg activation and centrosome formation is induced immediately after oviposition–independent of fertilization. Hence, a transition to parthenogenesis may involve a relatively small change–‐and thus occur frequently–in haplodiploids (Engelstaedter 2008). Nevertheless, the extensive variation in the frequency of parthenogenesis among haplodiploids (Figs. 1, S1–S3) clearly shows that different sex determination systems are not necessary to explain the variation in the frequency of parthenogenesis among major taxa.

The incidence of polyploidy and endosymbiont‐induced parthenogenesis is much lower than commonly thought. We find that polyploidy is extremely rare in parthenogenetic haplodiploids. This shows that—at least in haplodiploids—polyploidy is not a common consequence of parthenogenesis. Parthenogenesis‐inducing endosymbionts are found in 42% of the cases, but this is almost certainly an overestimate due to publication bias. Convincing evidence supporting endosymbiont‐induced parthenogenesis is reported for several taxonomic groups, albeit in the vast majority of cases the causal endosymbiont remains unknown. There is a clear need for more studies that provide balanced evidence supporting or rejecting endosymbionts as causal agents for parthenogenesis (e.g., van der Kooi and Schwander 2014a; Monti et al. 2016), as well as detailed studies that characterize the endosymbiont species in systems where endosymbiont‐induced parthenogenesis is suggested.

An in‐depth comparative analysis of parasitoid and phytophagous wasps showed that parthenogens have more host species and wider geographical distributions than sexuals (Figs. 3, 4). These differences mimic the large distribution ranges and/or poleward expansions in self‐fertilizing (Grossenbacher et al. 2015) and asexual plants (Johnson et al. 2010). The extended distribution of parthenogenetic lineages toward the poles could stem from enhanced colonization abilities in these lineages, for example because parthenogenesis confers reproductive assurance [“Baker's law”; (reviewed by Pannell et al. 2015)] and/or because parthenogens have an advantage over sexual relatives in colder climates. For example, cold climates can generate colonization‐extinction cycles (i.e., strong reductions in population size during extreme winters), which can lead to mate limitation and inbreeding in sexuals (Haag and Ebert 2004). Given that mate limitation and inbreeding will never be a problem for parthenogenetic females, parthenogenetic lineages may be more successful in (re)colonizing habitats after extreme winters. Ecological niche width and geographic distribution range are likely to be interrelated, but the relative importance of either remains currently unknown.

The wider ecological niches and geographical ranges found in parthenogens only partially evolved after the transition to asexuality. Sexual sister‐species–that have a shared ancestor with the parthenogenetic lineage–exhibit relatively large ecological niches and distribution ranges, as compared to sexuals for which no related parthenogenetic species is known (Figs. 4, S6). A similar pattern was suggested to occur in (diploid) parthenogenetic scale insects (Ross et al. 2013), and emphasizes that large population sizes of sexuals are paramount in the origin and/or evolutionary success of derived parthenogenetic lineages. Large population sizes should favor the evolution of parthenogenesis because more mutants capable of parthenogenesis are expected in species with larger population size. Parthenogens with large population and range sizes are also less prone to extinction than those with small populations (e.g., Otto and Barton 2001). We also found weak evidence that parthenogenesis emerges more frequently in species with small body sizes, which generally also have larger populations (Supplementary Information).

Finally, the development of this database opens perspectives on future comparative studies on the evolution of sex. Of particular interest would be to develop parthenogenetic species lists for groups with other sex determination systems.

### AUTHOR CONTRIBUTIONS

C.J.v.d.K. and T.S. designed the study; C.J.v.d.K. developed the species database with input from all authors; C.M.D. developed the tool to automatically obtain data from the Universal Chalcidoidea Database; C.J.v.d.K. and C.M.D. performed the comparative analyses; C.J.v.d.K. and T.S. wrote the manuscript with input from all authors.

Associate Editor: K. Lythgoe

## Supporting information


**Figure S1**. Frequency of parthenogenesis in Chalcidoidea. Phylogeny from Heraty*, et al*. (1). Total number of species per family was taken from Noyes (2).
**Figure S2**. Frequency of parthenogenesis in Symphyta. Phylogeny from Klopfstein, *et al*. (3) and the total number of species was taken from Taeger and Blank (4). 14 families with fewer than 80 species documented in total were excluded (these all had 0 parthenogens).
**Figure S3**. Frequency of parthenogenesis in Thysanoptera. Phylogeny after Buckman, *et al*. (5) and species totals were taken from Mound (6).
**Figure S4**. Polyploidy in parthenogenetic haplodiploids. Based on studies with chromosome counts 4% (2 in 50) are polyploid. When species with endosymbiont‐induced parthenogenesis (that very likely are diploids, see text) are included, only 2% (2 in 98) is polyploid.
**Figure S5**. Distribution ranges of sexuals and parthenogens (in absolute values). Left panels: pairwise analyses, right panels: analyses incorporating all information from the database.
**Figure S6**. Ecological niche width and distribution ranges for sexual species with different relatedness to parthenogens. Sexual species closely related to parthenogens (i.e. their sexual sister‐species) have more host species and wider geographic ranges than their outgroup (i.e. sexual species within the same genus, for which no parthenogenetic sister‐species are known). Dashed lines refer to related parthenogenetic species; p‐values refer to the sister species *versus* outgroup comparison. For the complete comparison between parthenogens and their sexual sister species, see Figure 4 (left panels) of the main text.
**Figure S7**. Body sizes of sexual and parthenogenetic Chalcidoidea.
**Table S1**. Genera with clear evidence of endosymbiont‐induced parthenogenesis in one or more species.

Supporting Information

Supporting Information

Supporting Information

Supporting Information

Supporting Information

Supporting Information

Supporting Information
