# Supplementary material for: Evolution and comparative ecology of parthenogenesis in haplodiploid arthropods
Source: Evol Lett. 2017 Nov 9;1(6):304–16. doi: 10.1002/evl3.30 (PMC6121848; doi:10.1002/evl3.30)
Supplement: Supplementary file 1 — Figure S1. Frequency of parthenogenesis in Chalcidoidea. Phylogeny from Heraty, et al. (1). Total number of species per family was taken from Noyes (2). Figure S2. Frequency of parthenogenesis in Symphyta. Phylogeny from Klopfstein, et al. (3) and the total number of species was taken from Taeger and Blank (4). 14 families with fewer than 80 species documented in total were excluded (these all had 0 parthenogens). Figure S3. Frequency of parthenogenesis in Thysanoptera. Phylogeny after Buckman, et al. (5) and species totals were taken from Mound (6). Figure S4. Polyploidy in parthenogenetic haplodiploids. Based on studies with chromosome counts 4% (2 in 50) are polyploid. When species with endosymbiont‐induced parthenogenesis (that very likely are diploids, see text) are included, only 2% (2 in 98) is polyploid. Figure S5. Distribution ranges of sexuals and parthenogens (in absolute values). Left panels: pairwise analyses, right panels: analyses incorporating all information from the database. Figure S6. Ecological niche width and distribution ranges for sexual species with different relatedness to parthenogens. Sexual species closely related to parthenogens (i.e. their sexual sister‐species) have more host species and wider geographic ranges than their outgroup (i.e. sexual species within the same genus, for which no parthenogenetic sister‐species are known). Dashed lines refer to related parthenogenetic species; p‐values refer to the sister species versus outgroup comparison. For the complete comparison between parthenogens and their sexual sister species, see Figure 4 (left panels) of the main text. Figure S7. Body sizes of sexual and parthenogenetic Chalcidoidea. Table S1. Genera with clear evidence of endosymbiont‐induced parthenogenesis in one or more species. [file EVL3-1-304-s001.docx]

**Supplementary figures**


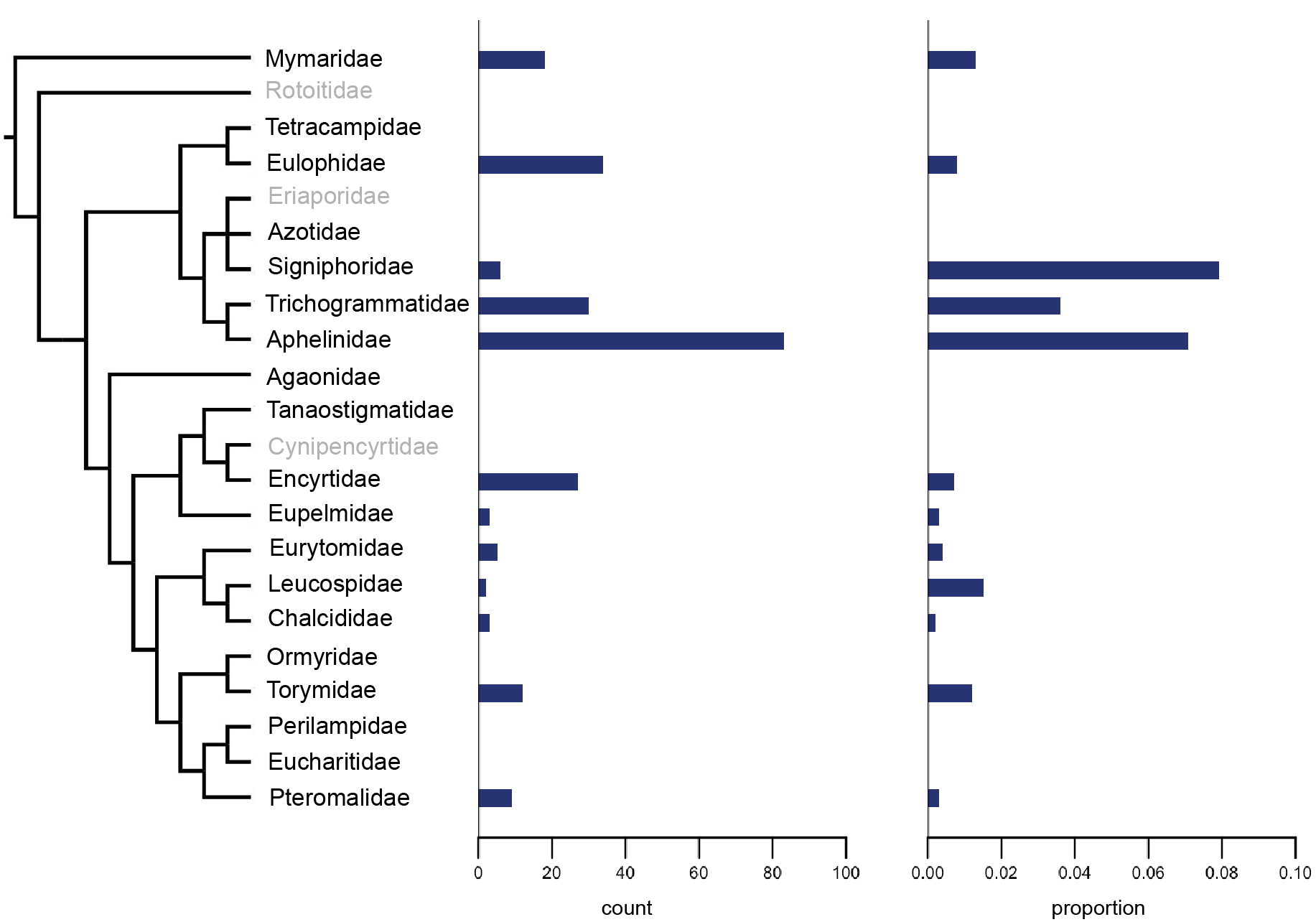


Figure S1. Frequency of parthenogenesis in Chalcidoidea. Phylogeny from Heraty*, et al.* (1). Total number of species per family was taken from Noyes (2).


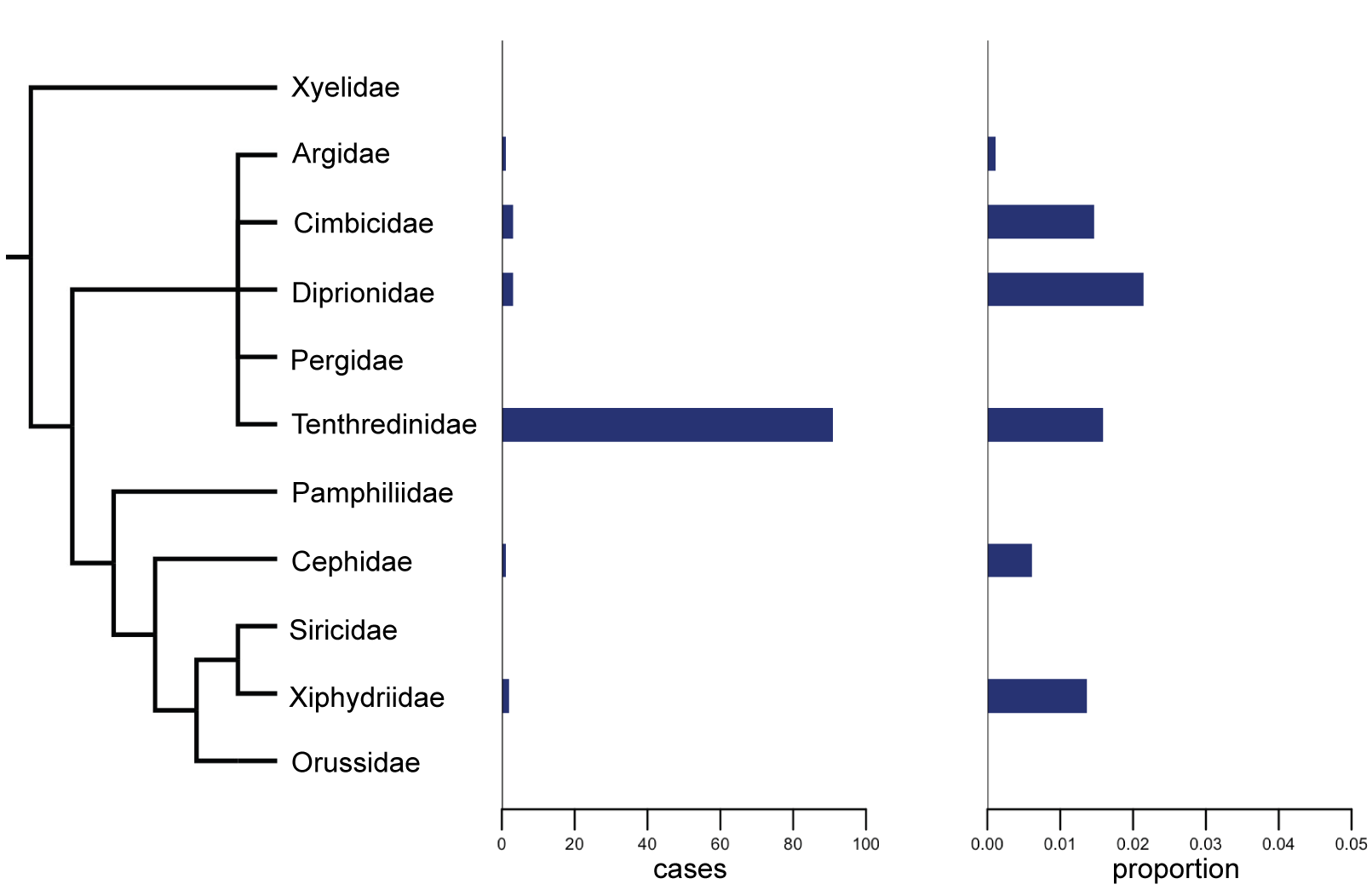


Figure S2. Frequency of parthenogenesis in Symphyta. Phylogeny from Klopfstein, Vilhelmsen, Heraty, Sharkey and Ronquist (3) and the total number of species was taken from Taeger and Blank (4). 14 families with fewer than 80 species documented in total were excluded (these all had 0 parthenogens).


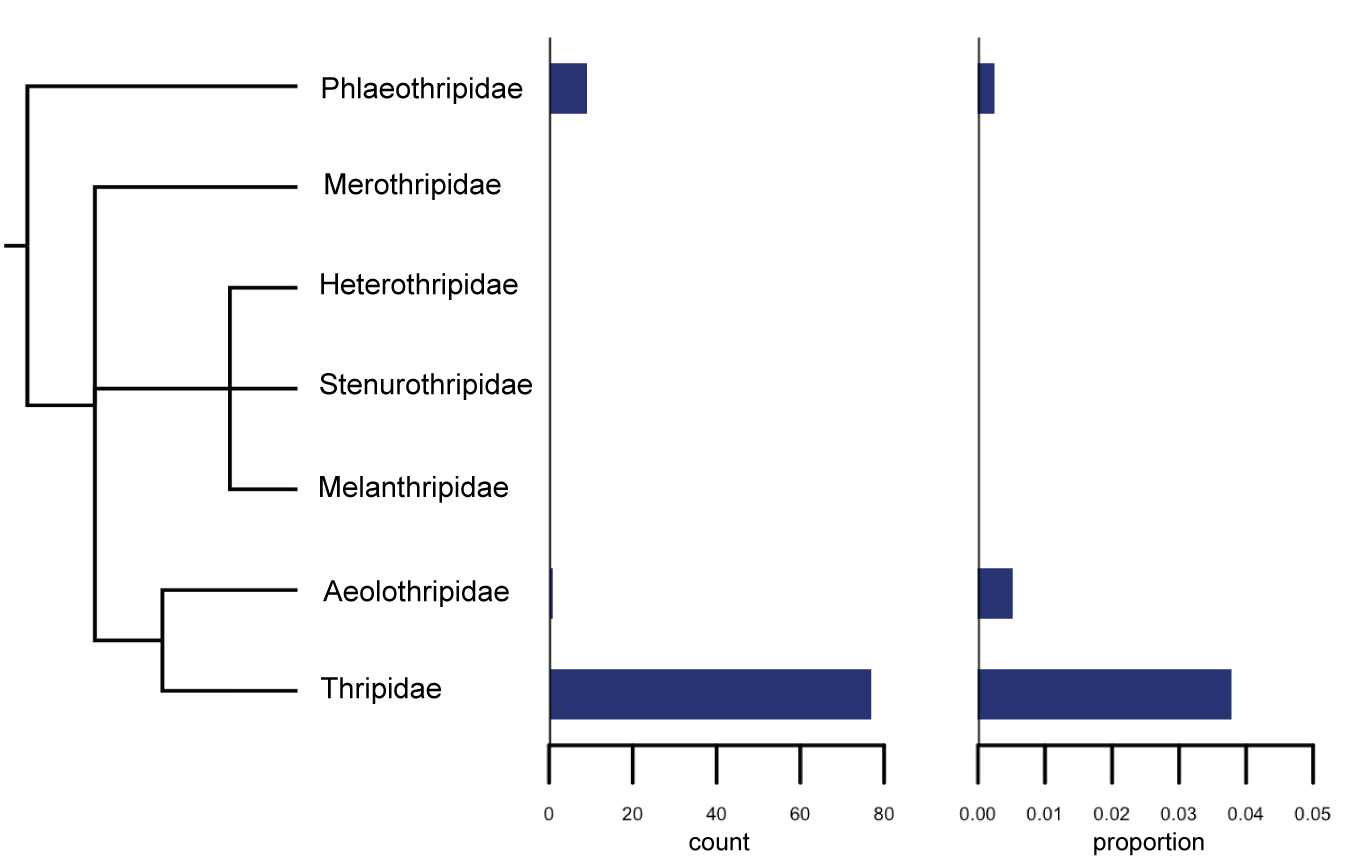


Figure S3. Frequency of parthenogenesis in Thysanoptera. Phylogeny after Buckman, Mound and Whiting (5) and species totals were taken from Mound (6). The very small families Uzelothripidae (1 extant putatively sexual species) and Fauriellidae (5 extant putatively sexual species) were excluded, because of unclear phylogenetic relatedness.


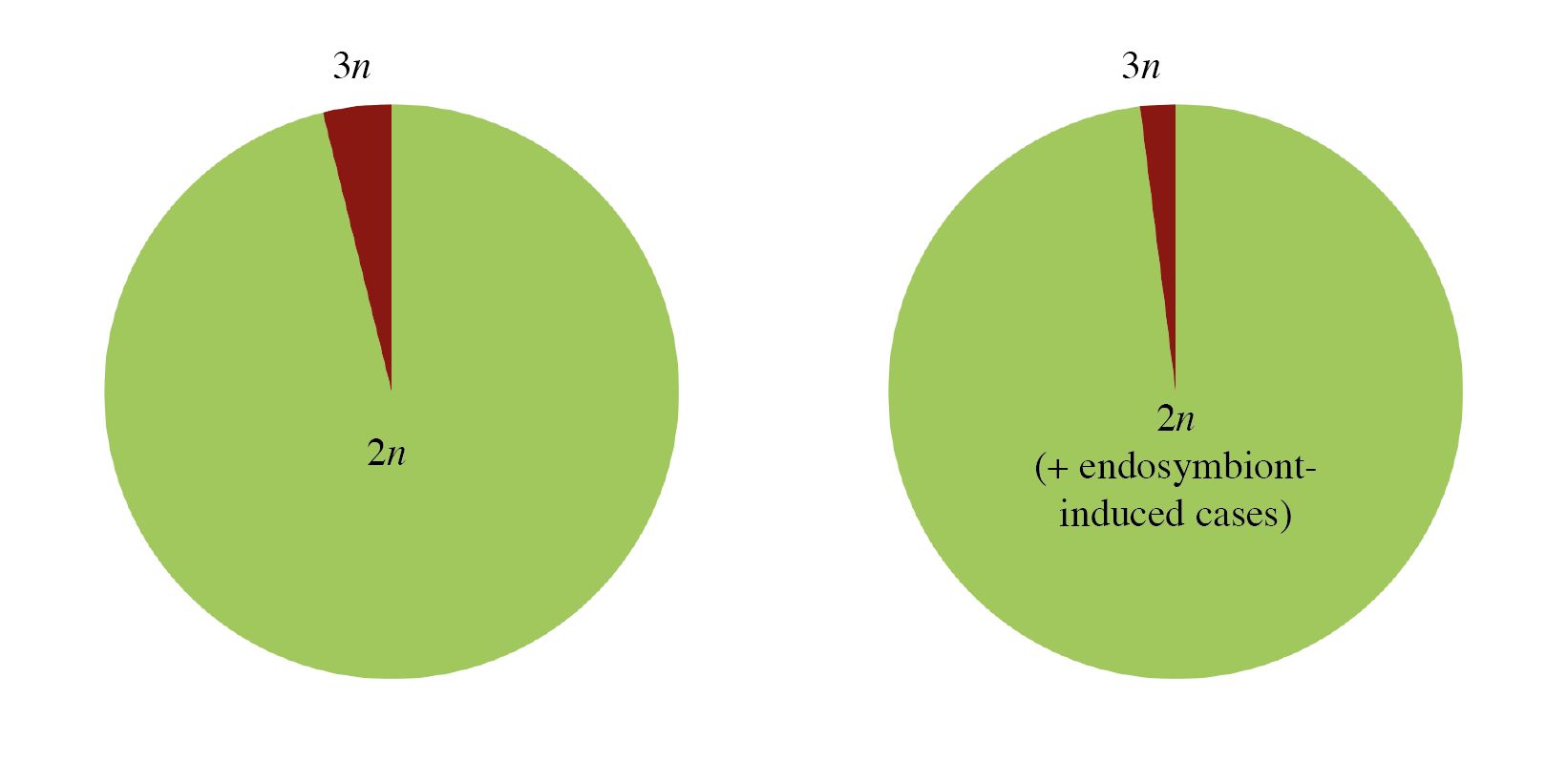


Figure S4. Polyploidy in parthenogenetic haplodiploids. Based on studies with chromosome counts 4% (2 in 50) are polyploid. When species with endosymbiont-induced parthenogenesis (that very likely are diploids, see text) are included, only 2% (2 in 98) is polyploid.

Figure S5. Distribution ranges of sexuals and parthenogens (in absolute values). Left panels: pairwise analyses, right panels: analyses incorporating all information from the database.

Figure S6. Ecological niche width and distribution ranges for sexual species with different relatedness to parthenogens. Sexual species closely related to parthenogens (i.e. their sexual sister-species) have more host species and wider geographic ranges than their outgroup (i.e. sexual species within the same genus, for which no parthenogenetic sister-species are known). Dashed lines refer to related parthenogenetic species; p-values refer to the sister species *versus* outgroup comparison. For the complete comparison between parthenogens and their sexual sister species, see Figure 4 (left panels) of the main text.

Figure S7. Body sizes of sexual and parthenogenetic Chalcidoidea.

Table S1. Genera with clear evidence of endosymbiont-induced parthenogenesis in one or more species.

| **Order** | **Family** | **Genus** |
| --- | --- | --- |
| Hemiptera | Diaspididae | *Aonidiella* |
| Hymenoptera | Aphelinidae | *Aphelinus, Aphytis, Encarsia, Eretmocerus* |
|  | Braconidae | *Asobara* |
|  | Diprionidae | *Diprion* |
|  | Encyrtidae | *Anagyrus, Coccidoxenoides, Diaphorencyrtus, Habrolepis, Ooencyrtus, Plagiomerus, Trechnites* |
|  | Eulophidae | *Galeopsomyia, Neochrysocharis, Pnigalio* |
|  | Figitidae | *Gronotoma, Hexacola, Leptopilina, Odontosema* |
|  | Mymaridae | *Anagrus* |
|  | Platygastridae | *Telenomus* |
|  | Pteromalidae | *Muscidifurax* |
|  | Signiphoridae | *Signiphora* |
|  | Tenthredinidae | *Pristiphora* |
|  | Torymidae | *Megastigmus* |
|  | Trichogrammatidae | *Trichogramma* |
| Thysanoptera | Aeolothripidae | *Franklinothrips* |
|  | Thripidae | *Aptinothrips, Hercinothrips, Taeniothrips* |
| Trombidiformes | Tetranychidae | *Bryobia* |

**References**

1. Heraty JM*, et al.* (2013) A phylogenetic analysis of the megadiverse Chalcidoidea (Hymenoptera). *Cladistics* 29(5):466-542.

2. Noyes JS (2016) Universal Chalcidoidea Database. World Wide Web electronic publication. <http://www.nhm.ac.uk/chalcidoids>.

3. Klopfstein S, Vilhelmsen L, Heraty JM, Sharkey M, & Ronquist F (2013) The hymenopteran tree of life: evidence from protein-coding genes and objectively aligned ribosomal data. *PLoS One* 8(8):e69344.

4. Taeger A & Blank S (2011) ECatSym - Electronic World Catalog of Symphyta (Insecta, Hymenoptera). Program version 3.10, data version 38. Digital Entomological Information, <http://www.sdei.de/ecatsym/index.html>. (Müncheberg).

5. Buckman RS, Mound LA, & Whiting MF (2013) Phylogeny of thrips (Insecta: Thysanoptera) based on five molecular loci. *Systematic Entomology* 38(1):123-133.

6. Mound LA (2013) Order Thysanoptera Haliday, 1836. In: Zhang, Z.-Q.(Ed.) Animal Biodiversity: An Outline of Higher-level Classification and Survey of Taxonomic Richness (Addenda 2013). *Zootaxa* 3703(1):49-50.
