# Supplementary material for: Evolution and comparative ecology of parthenogenesis in haplodiploid arthropods
Source: Evol Lett. 2017 Nov 9;1(6):304–16. doi: 10.1002/evl3.30 (PMC6121848; doi:10.1002/evl3.30)
Supplement: Supplementary file 2 — Supporting Information [file EVL3-1-304-s002.docx]

| The statistical analyses were performed with a series of generalized linear mixed models with permutations on the relevant datasets. |
| --- |
|  |

| The reproductive modes are permuted randomly in each pair over 10000 simulations (nboot parameter) to generate an empirical distribution of z-values. The observed z-value is then compared with this distribution. |
| --- |
|  |

|  |
| --- |

|  |
| --- |
|  |

| library(nlme); library(lme4); library(parallel) |
| --- |
|  |

|  |
| --- |
|  |

|  |
| --- |

| nboot <- 10000 # Number of permutations |
| --- |
|  |

| variable = "host_spp" # Variable to be tested |
| --- |
|  |

| #################################################### |
| --- |
|  |

| # Randomize the mode (sex, asex) within a genus |
| --- |
|  |

| n.pairs <- length(levels(data$pair)) # number of genera |
| --- |
|  |

| l.genus <- as.vector(table(data$pair)) #list w/ number of species per genus |
| --- |
|  |

| fmla <- as.formula(paste(variable,"~ mode + (1\|genus/pair)",sep=" ")) |
| --- |
|  |

| m_host <- glmer(fmla, data = data,family = "poisson") |
| --- |
|  |

| zobs <- coef(summary(m_host))[2, "z value"] |
| --- |
|  |

|  |
| --- |
|  |

|  |
| --- |
|  |

| random_test <- function(x,y) { #x: data, y:genus |
| --- |
|  |

|  |
| --- |
|  |

| pair_name <- subset(x, pair == y)$pair |
| --- |
|  |

| species_name <- subset(x, pair == y)$species |
| --- |
|  |

| var <- subset(x, pair == y)[,variable] |
| --- |
|  |

| genus_name <- subset(x, pair == y)$genus |
| --- |
|  |

|  |
| --- |
|  |

| # Sample without replacement |
| --- |
|  |

| random_mode <- sample(subset(x, pair == y)$mode) |
| --- |
|  |

|  |
| --- |
|  |

| # Return a partial data frame (for each genus) |
| --- |
|  |

| return(data.frame(pair_name, genus_name, species_name, var, random_mode)) |
| --- |
|  |

| } |
| --- |
|  |

|  |
| --- |
|  |

| #################################################### |
| --- |
|  |

| # For each genus, run the random_test() function. |
| --- |
|  |

|  |
| --- |
|  |

| zval_model <- function(data, n.pairs,count=F){ |
| --- |
|  |

|  |
| --- |
|  |

| # Complete data frame initialization. |
| --- |
|  |

| ref.distri <- data.frame(x= character(0), y= character(0), z = character(0)) |
| --- |
|  |

|  |
| --- |
|  |

| for (t in 1:n.pairs) { |
| --- |
|  |

|  |
| --- |
|  |

| # Sub data frame (for each genus). |
| --- |
|  |

| part_distri <- random_test(data, levels(data$pair)[t]) |
| --- |
|  |

|  |
| --- |
|  |

| # Concatenation of each sub data frames. |
| --- |
|  |

| ref.distri <- rbind(ref.distri, part_distri) |
| --- |
|  |

| } |
| --- |
|  |

|  |
| --- |
|  |

| #print(ref.distri) |
| --- |
|  |

|  |
| --- |
|  |

| # Model |
| --- |
|  |

| if(count){ |
| --- |
|  |

| m1 <- glmer(var ~ random_mode + (1\|genus_name/pair_name), data = ref.distri,family = "poisson") |
| --- |
|  |

| st <- "z" |
| --- |
|  |

| } else{ |
| --- |
|  |

| m1 <- lmer(var ~ random_mode + (1\|genus_name/pair_name), data = ref.distri) |
| --- |
|  |

| st <- "t" |
| --- |
|  |

| } |
| --- |
|  |

| print(coef(summary(m1))) |
| --- |
|  |

| return(coef(summary(m1))[2, paste0(st," value")]) # Return zvalue |
| --- |
|  |

}
